# The Incremental Growth of Data Infrastructure in Ecology (1980–2020)

**DOI:** 10.1002/ece3.70444

**Published:** 2024-12-10

**Authors:** Karen S. Baker, Florence Millerand

**Affiliations:** ^1^ Department of Information Sciences University of Illinois, Urbana‐Champaign Urbana Illinois USA; ^2^ Département de Communication Sociale et Publique Montréal Quebec Université du Québec à Montréal Latin Quarter Montreal Canada

**Keywords:** data infrastructure, data management, incremental growth, information system trajectory, open data, open science

## Abstract

After decades of growth, a research community's network information system and data repository were transformed to become a national data management office and a major element of data infrastructure for ecology and the environmental sciences. Developing functional data infrastructures is key to the support of ongoing Open Science and Open Data efforts. This example of data infrastructure growth contrasts with the top‐down development typical of many digital initiatives. The trajectory of this network information system evolved within a collaborative, long‐term ecological research community. This particular community is funded to conduct ecological research while collective data management is also carried out across its geographically dispersed study sites. From this longitudinal ethnography, we describe an Incremental Growth Model that includes a sequence of six relatively stable phases where each phase is initiated by a rapid response to a major pivotal event. Exploring these phases and the roles of data workers provides insight into major characteristics of digital growth. Further, a transformation in assumptions about data management is reported for each phase. Investigating the growth of a community information system over four decades as it becomes data infrastructure reveals details of its social, technical, and institutional dynamics. In addition to addressing how digital data infrastructure characteristics change, this study also considers when the growth of data infrastructure begins.

## Graphical abstract

After four decades of growth, a research community's network information system and data repository were transformed to become a national data management office and infrastructure for the environmental sciences. Developing functional data infrastructure is key to support of ongoing Open Science and Open Data efforts. In contrast with the more typical top‐down planning for many large‐scale digital initiatives, investigation of the evolution of this community's information system over the long term, reveals an incremental growth approach to understanding the dynamic interdependence of social, technical, and organizational components of a data ecosystem.

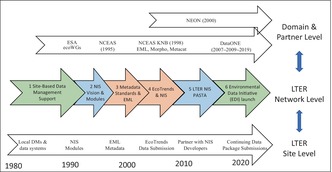

## 1 Introduction

We investigate the growth of an information system and its role as data infrastructure for a research community responding to continuing change in the digital era. The Long‐Term Ecological Research (LTER) program's development of a Network Information System (NIS) for managing long‐term data provides an example of supporting scientific research while addressing the increasing demands of data work.

Information systems are central to contemporary Open Science efforts to ensure the transparency of science. Open Science includes Open Data that aims to assemble data and make it accessible. This data effort depends upon the development of individual and collective data practices as well as data infrastructure. Such efforts benefit from ethnographic studies of research environments with their many significant differences in logistics, methods, and interests as well as in support structures, data arrangements, and community assumptions. These differences give rise to the variety of data infrastructures available today (Pomerantz and Peek 2016; Hampton et al. 2015; Borgman 2015; Kitchen 2014). Care is needed to avoid considering only early technology‐centric designs that may preclude community colearning, user participation, and preservation of local knowledge as growth occurs. National and international reports describe Open Science and Open Data as having goals such as facilitating data access and reuse (TRS 2012; EU 2016; OECD 2015; NAP 2018). Descriptions of existing digital arrangements contribute to understanding potential responses to Open Data mandates such as those from the U.S. Office of Science and Technology (OSTP 2013, 2022) and the National Science and Technology Council (NSTC 2022).

This case presents the opportunity to address three research questions:

- How does the growth of digital data infrastructure occur over time?
- What characteristics and data workers are associated with the evolution of an information system?
- When does the growth of digital data infrastructure begin?

This study provides some options for planning data management to achieve Open Data and makes a number of additional contributions relating to these research questions. First, using this case, we propose an Incremental Growth Model that describes an information system trajectory as a series of phases initiated by pivotal events, thereby capturing both the stability and change involved. Second, we identify major characteristics of each phase of an information system's growth. Third, rather than selecting a few snapshots in time, analysis of each phase provides key insights relating to the growth of data infrastructure over 40 years. Finally, to facilitate comparative studies, we outline this case's contextual conditions.

## 2 Background

To study a scientific research program as it grapples with data issues and data systems, we draw on Infrastructure Studies informed by Science and Technology Studies, an approach that ensures sensitivity to the complexity of a digital environment. In addition, Organizational Change Research contributes to the framework, which was developed to capture the broader context of events and major characteristics associated with an information system's trajectory. We begin by introducing the concepts of data infrastructure, continuing design, and pivotal events. To address data infrastructure growth, we also consider key issues associated with data work and data workers before introducing the LTER program.

### 2.1 Data Infrastructure

The term digital infrastructure is associated with computers, platforms, and software. In a National Science Foundation (NSF) report, the scope of infrastructure was broadened dramatically from a technically oriented understanding to include services and organizations as well as the people who design, develop, and facilitate (Atkins 2003). Subsequently, NSF reported on cyberinfrastructure as meeting the need for new kinds of organization and computing that would include planning for data and a data workforce (NSF CI Report 2007, 2019).

Infrastructure as conceptualized and characterized by Star and Ruhleder (1996) is a multifaceted concept referring to interrelated arrangements involving hardware, software, standards, procedures, practices, and policies together with support for human communication and capabilities. Research has highlighted the need to consider how infrastructure:

- Forms and accumulates over time in collaborative and iterative design (Edwards et al. 2007; Erickson and Sawyer 2019; Karasti, Pipek, and Bowker 2018).
- Ensures flexibility through configurations including an installed base, kernel, and/or gateways (Star and Ruhleder 1996; Hanseth 2010; Ribes 2014).
- Benefits from the development of standards (Edwards 2004; Millerand and Bowker 2009; Hanseth 2010).
- Represents a dynamic digital ecosystem (Mongili and Pellegrino 2014; Harvey, Jensen, and Morita 2017; Millerand and Baker 2020).
- Maintains resiliency via loose couplings and recouplings within and between elements, components, and systems (Winter et al. 2014; Misangyi 2016; Miller 2018; Orton and Weick 1990; Arango‐Vasquez and Gentilin 2021).
- Evolves into a variety of forms of knowledge infrastructure (Edwards et al. 2013; Bowker 2017; Borgman, Scharnhorst, and Golshan 2019).

Infrastructures today are considered complex phenomena that involve a collection of interacting digital systems and services (Bowker et al. 2010). Infrastructure from an information systems perspective, shifts from considering the alignment of digital systems to a broader view that takes into account the changing components of systems and subsystems supported by digital workers. Data Infrastructure Studies recognize the agency of humans and technologies separately and together: “infrastructures are shaped by multiple agents with competing interests and capacities, engaged in an indefinite set of distributed interactions over extended periods of time. The characteristics of infrastructure emerge out of these interactions” (Harvey, Jensen, and Morita 2017). To address infrastructural complexity, Hepsø, Monteiro, and Rolland (2009) present the notion of ecologies of e‐infrastructure within a business setting where six general categories of important events are identified, and Pollock and Williams (2010) develop the “Biography of Artifacts” concept using multi‐sited studies for comparative analysis of enterprise systems in business and health settings.

Infrastructure emerges when interacting systems of systems and people become an established part of a community's work. Many terms are used in referring to such digital arrangements including information infrastructure, research infrastructure, and cyberinfrastructure. We focus on data infrastructure “as one type of information infrastructure that supports science by facilitating individual and collective work with data (…) where it includes more than digital technologies as [it is] also constituted of individuals, organizations, routines, shared norms, and practices” (Millerand and Baker 2020). The interdependence of social, technical, and institutional facets of these systems results in a complexity often difficult to describe fully. In such an environment, it is critical to keep in mind that any single perspective on such phenomena will be partial.

### 2.2 Digital Data Infrastructure and Continuing Design

A linear approach was used in building early digital systems with all stages of development planned at project start. In time, such static planning has been found to be limited by a lack of user input and inadequate adaptive capabilities. An iterative design approach incorporating agile and user‐centered development avoids many such limitations through the use of dynamic planning including repeating cycles of design‐develop‐test. This kind of growth provides the time needed for learning between participants. Each cycle spurs the evolution and evaluation of software, applications, and systems. Iterative design ‐including methodologies such as participatory design (Simonsen and Robertson 2013), participatory infrastructuring (Bødker, Dindler, and Iversen 2017), collaborative design (Pipek, Karasti, and Bowker 2018), and meta‐design (Fischer and Ostwald 2002; Fischer and Herrmann 2011, 2015) – focuses on dynamic processes and interactions between designers, users, and their environment. Iterative design circles back to the concept of “continuing design” (Henderson and Kyng 1991) to emphasize developments both within a cycle and cumulatively over all the phases. The term “infrastructuring” emerged to underscore the active process of creating and maintaining infrastructure (Karasti and Baker 2004; Pipek and Wulf 2009; Pollock and Williams 2010; Karasti, Pipek, and Bowker 2018). It signals a move from the idea of infrastructure as a built object or a stable product to imagining an evolving entity (Hanseth 2010) or a process of growth sometimes referred to as “infrastructure in‐the‐making” (Edwards et al. 2007; Pipek, Karasti, and Bowker 2017).

In cases of complexity and change, the notion of sensitizing concepts is used (e.g., Ribes and Polk 2014). For the case under study here, two facets of change are central: sociotechnical and institutional. A sociotechnical facet draws attention to the design and use of digital data and systems by humans (Aken 2004; Salgado, Morel, and Vérilhac 2018). It refers to the intertwining of the social and technical arrangements or actions. We define the sociotechnical facet to be inclusive of situations in digital arenas where participants work at individual, project, and community levels, such as with the use of spreadsheets in an individual's lab or submission of data to a local data system. Arrangements and actions in larger‐scale arenas are also sociotechnical. They are designated institutional facets, however, to highlight their place in hierarchical structures, such as a national organization's actions or an agency's funding program arrangements.

### 2.3 Incremental Growth and Pivotal Events

When an object of study is a process, new approaches are needed. Organization Research has developed “event structure analysis” (Hak, Jaspers, and Dul 2013) while Science and Technology Research recounts how “both rhythms and events can disrupt, shape and reorient plans” (Steinhardt and Jackson 2014). In the development of digital systems subject to ongoing interactions and change, sequential periods can be identified as phases. Whereas a long‐term plan provides an overarching general mission, a short‐term plan lays out possible advances for the system and its products given resources at hand for the current phase. A phase initiated by a major pivotal event persists for a period of time, ending when another pivotal event takes place. A pivotal event is defined as a time‐bound situation with highly consequential potential (Cutcher‐Gershenfeld, Brooks, and Mulloy 2015; Cutcher‐Gershenfeld 2020). It is a precipitating event that represents a point at which opportunities occur and choices are made. The term incremental growth is used to describe the manner in which plans for a digital system unfold over time in phases.

Information Systems Research focuses on digital networks and their relations as key to understanding infrastructure and to establishing, maintaining, or increasing alignment of systems. In their study of digital infrastructure evolution from a configurational perspective, Henfridsson and Bygstad (2013) identify generative mechanisms as “causal structures that generate observable events”, thereby creating potential paths for infrastructural evolution. They categorize generative mechanisms as adoptive, innovative, scaling, or some combination of these resulting in new services. Adoption mechanisms lead to more coordinated participation, innovation enables change, and scaling mechanisms lead to modular growth. Pivotal events function as generative mechanisms that spur critical changes in the trajectory of an information system.

### 2.4 Data Work and Data Workers

The notion of data, with its tangled relations to information and knowledge, frequently proves elusive to define (e.g., NRC 1997; Beaujardière 2016; Leonelli 2015, 2016). Borgman (2015) reports concisely that “data are representations of observations, objects, or other entities used as evidence of phenomena for the purposes of research or scholarship.” The increasing diversity in kinds of data in terms of origin, methods, collection, use, and users makes the work associated with data difficult to classify. Indeed, recent observations and cases illustrate the importance of the product manager and the commonplace lack of regard for the role in large‐scale digital efforts (e.g., Pahlka 2023). The role of data manager, one type of product manager, may encounter such disregard even in a data‐oriented community. The LTER case, with its Open Data and data infrastructure efforts, holds the potential to add to Pahlka's notion of “bottom‐up” or support for the “people in the trenches.”

Data work is defined as an effort applied to data that may involve heterogeneous data activities occurring in diverse settings that are carried out by humans and technologies separately and together (Baker 2017). For data workers today, data work roles are frequently force fit into earlier job classification systems that are inadequate for the wide range of data skills, tasks, and responsibilities now associated with research. Over the last decade, Human Resource Offices within universities (e.g., UCSD n.d.) and staff within libraries (e.g., Cox and Corrall 2013) have begun to address digital data roles. Whether expanding old job profiles or developing new ones with updated job functions, these efforts often lag behind the rapid diversification of the digital data workforce. Despite emerging titles such as software engineer, data manager, and visualization specialist, data workers draw attention to the marked lack of career paths today.

Data work was once solely in the hands of researchers. Today data workers are specialists in digital environments advancing digital fluency with new vocabulary such as metadata, data package, data flow, database, and data stewardship. The Long‐Term Ecological Research (LTER) program (e.g., Baker et al. 2000; Karasti and Baker 2004; Baker and Millerand 2010; Stafford 2021) provides an early example of establishing new data management practices and developing a data workforce at universities and other research organizations supported by federal funds. The role of data manager typically includes familiarity with the research process as well as selecting, implementing, maintaining, and advancing technology, software, applications, and/or services associated with data. This role is distinct from roles such as research assistant, data analyst, programmer, data scientist, software engineer, cyberinfrastructure specialist, or others concerned with computational systems and methods. The role is often situated within an environment that calls for skills in interpersonal communications and data care (Baker and Millerand 2007, 2010; Baker and Karasti 2018). From this position, data managers are able to introduce updates in data practices in addition to facilitating data organization and access. The role is sometimes recognized as a “sociotechnical bridge” for a community of researchers given the “boundary‐spanning” work needed across arenas (individual, project, network, and national) as well as across fields (data, informatics, software, computing, technology, and scientific research).

In this paper, several terms are used to describe those engaging in data work. The notion of “data workers” includes data managers and other data‐oriented participants. The role of the data worker at the first LTER sites was initially called “data manager” (DM), a designation used throughout this paper for clarity though the role was later renamed “information manager” within the LTER (Baker et al. 2000). The term “NIS‐data team” refers to LTER Network Office (LNO) software engineers and LTER site‐based data managers working together on a Network Information System with a high degree of awareness of the sociotechnical ramifications of data work. We use the software engineer category broadly to encompass a number of software, technology, and data specialists including developers, designers, and computing and networking professionals.

### 2.5 The LTER Program

#### 2.5.1 LTER History

The LTER program and its data efforts were informed by lessons learned a decade earlier in the International Biological Program **(**IBP). During the IBP from 1964 to 1974, ecological data was sometimes delivered as paper records to a set of offices distributed across the U.S. (Golley 1993; Aronova, Baker, and Oreskes 2010; Coleman 2010). The IBP goal to aggregate data was hindered by the lack of data practices, technologies, and management resources needed to support the challenges faced by the diversity of biological field projects. A LTER grant proposal to NSF to address this succeeded by focusing on the importance of long‐term data. The LTER program and its data management efforts have been supported by a number of visionary program leaders at NSF who recognized the need for and benefits of change. The NSF Division of Biological Infrastructure (DBI) provided initial guidance. LTER was also fortunate to have a number of insightful Principal Investigators who supported the new kinds of work associated with long‐term data.

The LTER program began as a set of six sites established in 1980, a time when databases were a new computational tool available to researchers in a few research arenas. Sites were funded as independent projects though expected to collaborate as a network. Through fieldwork at these ecological sites, researchers aimed to discern changes in each site's biome by collecting long‐term data. By 2017, the network had grown to 28 sites, each anchored at a university or other research center and consisting of an interdisciplinary team of scientists focused on a particular location. Unlike the highly centralized data facilities of the IBP, LTER in the beginning was configured to support site‐based efforts. An NSF mandate requiring each site to identify and support a local data manager set the stage for the program's work with data. Initially, sites developed local data practices and data systems that assembled data from the many project members at a site. Just as in ecology, where differences in ecosystems are fully appreciated and expected, the LTER sites developed understandings of their local “data ecosystem.” In the 1990s, when an LTER Network Office (LNO) was established, each site contributed to cross‐site ecological research as well as to network‐wide data work. Data managers had to learn how to balance site and network data activities (Baker and Karasti 2004; Karasti, Baker, and Millerand 2010). Their cross‐site communication and coordination of data was enabled by the early formation of an Information Management Committee (IMC) that was established as an LTER Network standing committee. The IMC became an active community of practice with data managers engaged from all the sites.

#### 2.5.2 LTER Contextual Conditions

Henfridsson and Bygstad (2013) work with the concept of contextual conditions to facilitate comparative analyses of case studies. Six LTER conditions describe the setting and arrangements within which the LTER Network Information System (NIS) evolved. They are summarized in Table 1 and discussed below.

**TABLE 1:** Contextual conditions describing the LTER case setting and arrangements for science and data.

**FIGURE 1:**
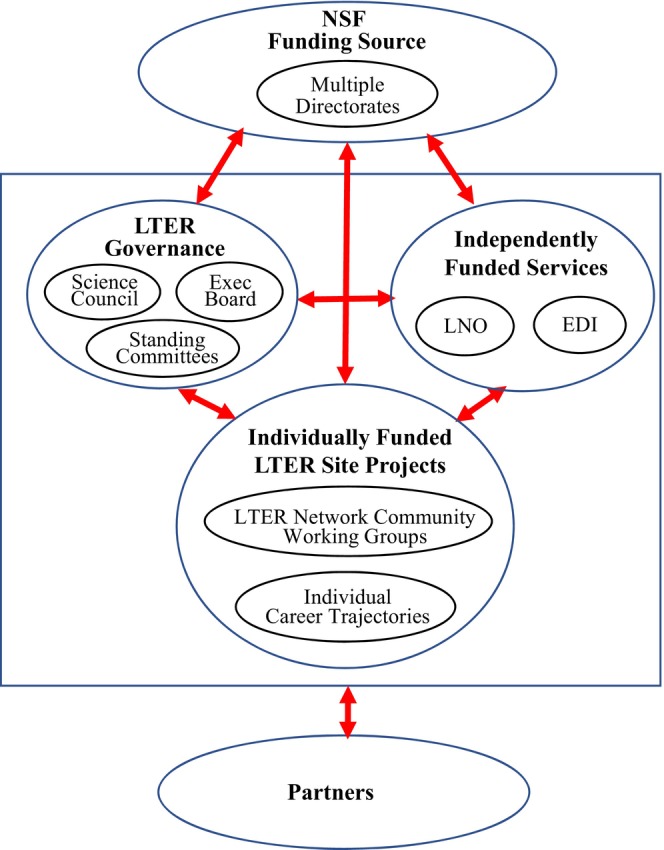
LTER communication structure: An ecosystem of reciprocal interactions (Drawing from Waide and Kingsland 2021, LTER https://lternet.edu/network‐organization/). This version shows arrangements after EDI was funded in 2016. Prior to this, the LTER Network Office was the only independently funded service.

1. *Program structure*: A science‐drive community established as a set of geographically distributed research sites, each funded independently to collaborate on a multi‐investigator project studying a designated biome, where these decentralized sites are joined together as a network with a shared vision, cross‐site research, and community governance with a network office providing supporting communication, and coordination.
2. *Long‐term funding*: Provisional, long‐term core funding supports the sites individually and the network while participants also pursue resources from other sources to augment existing capabilities and to fund new pursuits and/or equipment.
3. *Communication structure*: Five major components ‐ site projects, community governance, independently funded services (Network Office and Network Information System/EDI), partners, and NSF ‐ represent work arenas all interconnected by continuing reciprocal communications.
4. *Data management*: A data manager works at each site tasked with developing collective data practices, data management, and technical capabilities including juggling local data needs with network‐wide data activities associated with cross‐site research.
5. *Technology engagement*: Both site and network data workers take an agile, incremental, and integrative approach using iterative design with digital data work in support of scientific research.
6. *Community information system*: A network information system grew as a loosely coupled architecture with data‐oriented developers at the network office working closely with data managers and researchers at the sites, a collaboration that facilitates system use and data reuse.

(1) **Program Structure**: Each site studies an ecological biome over time, investigating local themes and five network‐wide core themes: “pattern and control of primary production, spatial and temporal distribution of populations selected to represent trophic structure, pattern, and control of organic matter accumulations, patterns of inorganic input and movements, and patterns and frequency of disturbance to the research site” (Callahan 1984). The LTER network carries out interdisciplinary research. It has characteristics associated with successful synthesis centers including active management, computing and informatics capabilities, flexibility, student and fellow support, diversity, and placing value on unstructured time (Baron et al. 2017).

(2) **Long‐Term Funding**: Sites were expected to take responsibility for the generation of long‐term data rather than the more typical 1‐ to 3‐year collections of data (Magnuson 1990). A new NSF funding model supported six‐year LTER funding cycles for each site with the expectation of a new cycle if their renewal proposal were accepted (Hobbie 2003). This new research model of long‐term funding was overseen by individuals in the often underappreciated position of NSF program director who over the years have played a significant role in both building and sometimes damaging LTER. These individuals together with program managers have been important as the network responded to change. For instance, the LTER community originally aimed to discern changes in their designated ecosystems. Studies initially focused on changes due to natural forces but soon included attention to human impacts (LTER Risser Report 1993). This envisioning of change due to human influences led in 1997 to the network expanding to include two urban sites.

(3) **Communication Structure**: The LTER communication structure is portrayed with five major components (Figure 1) that are discussed below.

- *Individually Funded LTER Site Projects*. Together the sites comprise the LTER Network community where interactions are actively supported via annual meetings, multi‐site working groups, newsletters, and mailing lists;
- *LTER Governance*. The LTER community is self‐governing with by‐laws that describe the work carried out by an Executive Board, Science Council, and standing committees, one of which is the Information Management Committee;
- *Independently Funded Services*. The LTER Network Office was initially the single independently funded service while network data management and digital systems were overseen by LNO as a standing committee and by NSF, which often led to complications in setting priorities; this changed in 2016 when network data management and NIS merged into the Environmental Data Initiative (EDI) to become an independently funded entity that partnered with LTER;
- *Partners*. Partners have added significantly to the community and have changed through the phases;
- *NSF Funding Source*. NSF is the agency that has funded LTER over the decades with support and input provided in time by multiple directorates.

This graphic is a simplification, reducing a complex web of interactions to a few components in order to portray the LTER communication structure as an ecosystem. It underscores the extent and dynamics of communications that tie together LTER participants. The figure does not intend to show decision‐making status or the respective influence of actors. Instead, it suggests that actions are negotiated, considered not just from one perspective but reconsidered, and informed by input from across the various components.

(4) **Data Management**: The designation of site‐based data managers raised and sustained awareness of data practices and data collection. Local site data work was continuously informed and influenced by data activities of researchers, data managers at other sites, and in time by development of the Network Information System. Annual funding for IMC meetings advanced understandings of data diversity, sharing of site‐based strategies, and approaches to reaching network goals. The shared experiences and assembly of information from all the sites spurred development of data sharing and engagement with the Network Information System. The close relationships of data workers with researchers at each site meant data activities remained aligned with everyday research practices and were tailored to facilitate data flow. Data workers associated with sites, LNO, and partnerships were recognized, resisted, and eventually trusted as change agents. Working within the research community, data workers in partnership with researchers became sustaining agents for Open Data efforts that enhanced scientific research and transformed the community culture.

(5) **Technology Engagement**: For a long‐term approach with minimal resources for technologies associated with data, LTER participants adopted practical approaches. The resulting development of new practices and procedures created time and opportunities for collective consideration and stepwise change while avoiding the burden of technology‐oriented grand promises and partnerships often accompanied by great expectations. The minimal funding approach prompted incorporation of short‐term goals and products within long‐term plans (Karasti, Baker, and Millerand 2010; Baker and Millerand 2010), creating a pace that enabled community participation in development of data standards (Millerand and Bowker 2009; Millerand et al. 2013), and giving rise to data strategies tailored to local interests (Millerand and Baker 2010).

(6) **Community Information System**: Imagining and enacting the community's network information system was done collaboratively by participants at the network office and the sites. Data management's sheltered position within a long‐term scientific research community shaped the growth of the data‐oriented digital realm. The IMC and a Network Information System Advisory Committee (NISAC) brought together data and technical specialists with researchers and LNO participants (Stafford 2021). The science‐driven community prompted and reviewed plans for data and computing via the IMC and NISAC. Negotiated understandings within the IMC sometimes were presented in the form of best practices (e.g., LTER EML Best Practices for LTER Sites 2004).

## 3 Methods

We use qualitative methods for this ethnography of infrastructure growth for a large community composed of individuals with differing viewpoints. Closely related to this case are studies at multiple locations over many years from which Pollock and Williams (2009, 2010) developed the notion of “strategic ethnography” for studying infrastructural technologies. This approach to ethnography incorporates theoretically informed, multi‐site, longitudinal investigations necessary to gain insights into the how, what, and when of infrastructure growth.

While ethnographic methods are diverse, they make use of tools such as case studies, participant‐observer roles, and quotes to capture participant voices. We worked alongside community members and also joined as genuine participants in the design process. From this “in house” position, we found interactions with LTER participants highly informative. Though providing detailed knowledge of data activities, we were aware an insider role could introduce potential biases from a site‐based, data‐oriented view. Collaboration, colearning, and entanglements were part of this long‐term study. These are notions explored by Hahn et al. (2018) who focus on the specifics of infrastructure events and activities that prompt collective reflection.

The first author of the present study spent a number of years (1990–2011) as data manager at two LTER sites and was active in the all‐site IMC as well as in working with the LTER NIS‐data team and NISAC. After the first decade, social scientists were invited to join in studying data management, data practices, and design of community information systems. This included the second author who studied LTER activities starting in 2004. Study continued thereafter with both more intense and less focused periods with the information management community, thereby blending over time both insider and outsider perspectives. From the second author's position within the LTER, a deep insider understanding of the data work and design activities developed. She witnessed and experienced many changes over time in collaboration with the first author.

Working with the LTER community included participating in meetings, conference calls, and workshops attended by a mix of researchers and data workers. We engaged in discussions sometimes at their request. Community member perspectives are presented in the following section using references and excerpts from their writings. This study has also drawn on digital documents maintained in personal files of the authors, the LTER community archive, and notes from sidebars at LTER data management meetings. The generation of field notes and field memos provided a record of immediate impressions and insights.

Our special relationship with LTER resulted in publications about data management as a community of practice (Karasti, Baker, and Halkola 2006; Karasti and Baker 2008; Baker and Millerand 2010), community metadata standard making as a process (Millerand and Bowker 2009), and the power and tensions involved in collaborations infused with “data troubles” (Millerand et al. 2013; Baker and Karasti 2018) including termination of a site (Kaplan, Baker, and Karasti 2021). Multi‐community, comparative studies that include LTER have provided further insights (Karasti and Syrjänen 2004; Ribes and Finholt 2007, 2009; Mayernik, Batcheller, and Borgman 2011; Ribes 2014).

In addition to information drawn from document analysis and previous studies, we also rely on recent interviews and informal meetings. In the last 6 years, our study conducted in‐person, semi‐structured interviews typically of 1.5 h within the LTER community. These included: researchers (4), data managers (18), information technologists (4) and program managers (2) from LTER sites, the LTER network, and NSF. These interviews focused on questions of design as well as data practices and infrastructure growth. One annual meeting of data managers in 2017 was filled with discussions about change by participants from new sites, mature sites, and terminated sites. Several in‐depth interviews, with one information technologist spurred our approach to seeing the development of the LTER NIS in phases.

Analysis began with open coding of transcriptions of audio‐recorded interviews and meetings. A number of synthetic timelines, overviews, and diagrams were created. Data triangulation was used to bring these materials together. We focused on the dual themes of data management and the Network Information System. In aiming to capture the 40‐year NIS trajectory, we developed, reworked, and reconfigured tables of category codes to identify major characteristics of significant events in the development of the LTER NIS.

Sampling across stakeholders is uneven over the period of this study, a limitation ameliorated by the review of historical reports and other documents. Additional interviews with information technologists and researchers would have improved the study. Another shortcoming is the loss of complexity and richness in order to convey the material within the length limitations of a journal article. Many important activities and events have been eclipsed.

## 4 Growth of the LTER Network Information System

**FIGURE 2:**
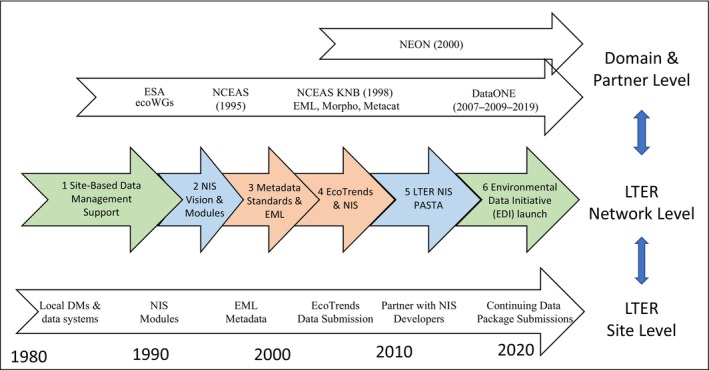
The six phases of the LTER Network Information System (NIS) trajectory are labeled “LTER Network Level.” Below this level are data activities at the “LTER Site Level” and above are related activities at the “Domain & Partner Level.” The phase colors highlight: Green, scaling prompts from external sources (see Section 5.1.6); Blue, internal partnering (see Section 5.1.4); and Red, external partnering (see Section 5.1.5).

The Long‐Term Ecological Research (LTER) Network program provides a unique example illustrating the evolution of an information system. The conceptual framing for our analysis draws on the notion of incremental growth. We show the growth of the LTER NIS as a sequence of six relatively stable phases within a multi‐level environment (Figure 2). For our research questions, these phases address the “how” of infrastructure growth.

Although many activities occur during a phase, our focus is on pivots that bring about a major change in data arrangements. Table 2 lists six major pivots and provides some context for each phase in the form of a brief timeline with dates of activities and digital products. The phases identified for the LTER NIS are described below. Each pivot initiates a phase featuring particular data work arenas where joint experiences and discussions occur. Below each phase is discussed together with changing community assumptions and the digital context with other efforts.

**TABLE 2:** Each pivot in the NIS trajectory is identified by a phase name and its focus. Dated events are listed to provide context followed by the approximate duration of each phase.

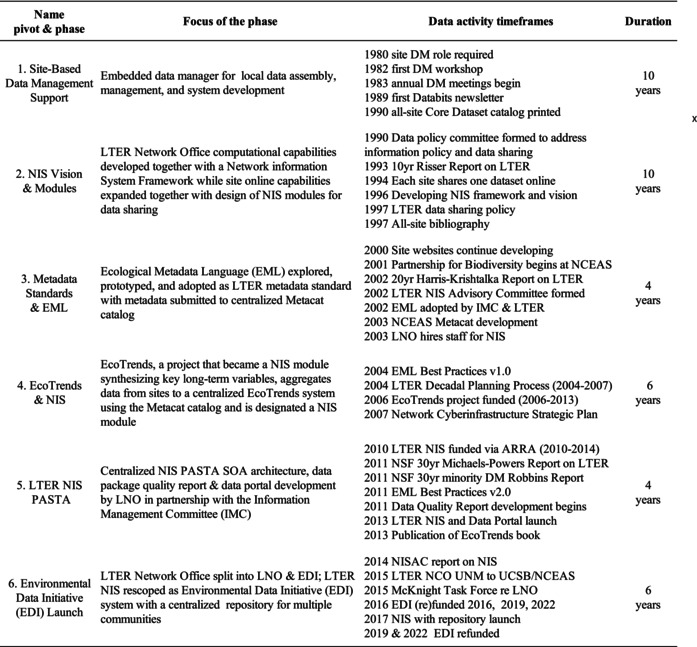

### 4.1 Phase 1: Embedding Data Management at LTER Sites (1980–1990)

The role of data manager was expected to appear as a line item in each site's budget. Naming of the data management position provided significant visibility to data‐related work. This role was a major change for research projects in 1980. With the prospect of continuing but limited funding for LTER from NSF, resources were stretched in supporting both site‐based collaborative research and the long‐term collection of field data. Tensions developed relating to supporting a data manager. Ecologists' exposure to technology in the field or lab often made advances easier to accept when they related to collecting the data rather than managing the data collectively for future data reuse. Traditionally researchers' primary accountabilities were related to their own research, not to external data standards, metadata creation, and data sharing (Mayernik 2017; Baker and Mayernik 2020). Researchers were trained in collecting and using their own data. Data managers themselves were grappling with identifying the responsibilities of data work. Annual meetings and monthly video conferencing of the all‐site IMC strengthened cross‐site ties with other data managers. Early work included physical artifact collections such as specimens, formatted data sheets, and print data reports. The creation of digital databases fueled growth of site computational capabilities. Assembly of data often went hand in hand with development of site‐based data systems. This experience positioned data managers as data champions able to identify and articulate data management issues at the opening of the 21st century with developments in digital technologies.

Collective data management was received as an unfunded expectation by LTER researchers though small additional funds for data management were made available intermittently. This required data workers to collaborate closely with site leaders to design and document data needs in small proposals to NSF that generally avoided the quick fixes often promised by high‐cost technologies. In working closely with researchers who generate the data, data managers emerged as a data workforce in sync with local research. Eventually data managers were able to look past the researchers' traditional horizon of data use for grant requirements and individual career advancement, to see new responsibilities of planning for data *reuse* by others.

During this NIS phase, the community assumption that “Data is best managed by individual researchers” shifts to “Data for multi‐investigator research efforts benefits from continuing development of project data management and data services.” This assumption shows recognition of the need for new ways of thinking about “data sets” by researchers (Franklin, Bledsoe, and Callahan 1990) and of the responsibilities that emerged for the role of data manager (e.g., Ingersoll, Seastedt, and Hartman 1997). At a time when the majority of researchers managed their own data, the LTER NIS trajectory Phase 1 raised awareness of the diversity of data and of managing data collectively. As researchers began asking longer‐term and larger‐scale research questions (Magnuson 1990; Swanson and Sparks 1990), the data managers were taking steps that would enable addressing their own longer‐term and larger‐scale questions about data issues including Open Data.

During this period, the U.S. Joint Global Ocean Flux Study (JGOFS, 1989–2005) was another early field research program that developed program‐oriented data management. Though the JGOFS centralized development differed from that of the LTER NIS trajectory, a JGOFS data office and repository was transformed in 2006 into a national Biological‐Carbon Oceanography‐Data Management Office (BCO‐DMO, https://www.bco‐dmo.org) for ocean sciences. It was funded by the Division of Ocean Sciences in the NSF Geosciences Directorate (Glover et al. 2006; Baker and Chandler 2008; BCO‐DMO 2013). BCO‐DMO continues to work today with oceanographic investigators via a central Data Management Office.

### 4.2 Phase 2: Envisioning a Distributed LTER Network Information System (1990–1999)

By 1990, internet connectivity brought dramatic change in terms of information access and data expectations. LTER published a Catalog of Core Data Sets in print form (Michener, Miller, and Nottrott 1990) with information about one dataset per page. Data accessibility was frequently noted as “digital, tape.” Due to rapidly evolving capabilities, however, electronic versions of a personnel directory and a Core Data Set Catalog were available at LNO by 1991 (Brunt 1998). Soon after, a NIS Interoperability Framework for a distributed data system was developed that depended on LNO computational technology as well as on specifications developed for harvest, exchange, metadata, indexing, and interfacing. All sites lacking digital capabilities were given critical support by LNO and IMC.

Making the data itself publicly available began network‐wide with an NSF prompt to LTER. In 1994, LTER leaders agreed that each site would make one dataset available on their site's webpages as a demonstration. Slowly the number of shared datasets online grew. As network planning and an increase in cross‐site research activities added to the interest in compiling data from all the sites (Johnson et al. 2010; Collins 2021), the vision for a Network Information System advanced. Development began with data managers at different sites and combinations of sites designing and hosting data modules including a climate database (Henshaw et al. 1998), a site description directory (Baker et al. 2000), and then a digital Data Table of Contents or DTOC (Porter 2010). DTOC was a precursor to updated catalog efforts in Phases 3 and 5. Leveraging the LTER Network Office technical resources, some modules were migrated to LNO. From prototyping work on NIS modules, data managers learned critical lessons about the little‐recognized work of “local enactment,” that is, about the sociotechnical activities required to align existing local data practices and systems with network applications (Millerand et al. 2005).

During this NIS phase, the community assumption that “Data can be stored using individual and laboratory storage devices” shifts to “Data from multiple sources can be brought together for sharing and reuse via submission to coordinated information system modules.” While a few researchers in Phase 2 envisioned some standardized approaches to network‐wide data handling (e.g., Franklin, Bledsoe, and Callahan 1990), others gained experience from the development of local data practices and data systems. In contrast to top‐down designs, the data managers' first approach to cross‐site data aggregation was to create data modules themselves since no money was budgeted for this (e.g., Henshaw et al. 1998; Baker et al. 2000). Participants from all the sites agreed to populate these modules, thus launching network information sharing and eventually prompting the development of data‐sharing policies, first at individual sites and eventually unified at the network level (Porter 2010). This phase demonstrates the LTER sites moving forward together given their collective involvement in the “how” of assembling centralized information.

During this period, access to the World Wide Web led many data managers in the 1990s to become their site's webmaster who organized online site content. This enabled sites to post project information, thereby contributing to site self‐awareness and eventually to making data available. In reviewing the web pages of other sites over time, researchers were exposed to a variety of materials, visualizations, and categories of information. These websites also enhanced site identity by prompting lively discussions about presentation of a site's research priorities and data priorities.

### 4.3 Phase 3: Partnering on Metadata and a Metadata Catalog (2000–2004)

From 1995, advances in computing, technology, and communication had increasing impacts on scientific work. Those managing data were contending with an array of instruments, procedures, documents, formats, and analyses. In assembling data from multiple sources, the need to annotate datasets with descriptive information called metadata became evident. Each site developed metadata that provided highly structured information including basics such as the dataset title. As the number of datasets increased, the metadata enabled sorting, discovering, and access to data.

LTER's work on metadata began in conjunction with the Ecological Society of America's Future of Long‐Term Ecological Data Committee (Michener et al. 1997), a committee with LTER members. NCEAS was active in supporting working groups of ecologists including LTER scientists, Software engineers at NCEAS were important partners for LNO and LTER data managers in development of data tools and data storage services as well as the Ecological Metadata Language (EML). After extensive work with NCEAS on metadata file formats, LTER adopted EML as the LTER metadata standard in 2002. Although aggregating data in a standard form had created difficulties earlier with the first LTER all‐site bibliography at LNO (Chinn and Bledsoe 1997), it took years to recognize that the movement of data from site systems to a community repository in a standardized form was a major issue rather than simply a local trouble (Millerand et al. 2013).

During this NIS phase, the community assumption that “Data can be described by metadata developed at a local level” shifts to “Data can be shared with larger audiences by mapping local metadata to metadata standards.” As LTER data managers aggregated data prior to Phase 3, they developed site‐specific metadata for use in local databases (Porter, Henshaw, and Stafford 1997; Millerand and Bowker 2009). Sites typically did not convert to EML locally but maintained their rich local metadata tailored to capture descriptive elements of a site's datasets for findability by local data system users. Sites eventually developed automated applications to map local metadata to EML prior to submission to NCEAS's metadata catalog (called Metacat) (Berkley et al. 2001). This centralized catalog and discovery system held metadata from all the LTER sites, initially with links to data stored at the sites. Local data systems served as site‐based gateway systems, eventually enabling automated machine‐to‐machine data submissions as well as the application of filters for data delivery. Questions that arose relating to the meaning of metadata entries for different communities led to LTER participants writing a customized LTER guide to the interpretation of descriptive tags for their community (LTER EML Best Practices for LTER Sites 2004). Thus the “how” of infrastructure for the LTER case involved partnering that expanded capabilities while documenting the particular needs of the LTER community.

During this period, partnerships contributed to formulating plans to advance data access. A Partnership for Biodiversity Informatics (PBI) working group was hosted by NCEAS in 2001 to pursue joint data interests, bringing together partners, including NCEAS, LNO, the San Diego Supercomputer Center (SDSC), and the Natural History Museum and Biodiversity Research Center at the University of Kansas, with the aim of giving access to information needed to sustain the earth. NCEAS led the multi‐institutional Knowledge Network in BioComplexity (KNB) project (Jones et al. 2006; Stafford 2021). Ecology as a whole benefited from the continuing efforts to raise awareness about data being both hard to find and hard to reuse. Although there were tensions, particularly associated with technical development, the role NCEAS played as an active center promoting data access was critical (Fegraus et al. 2005; Jones et al. 2006; Reichman, Jones, and Schildhauer 2011; Hampton et al. 2015).

### 4.4 Phase 4: EcoTrends and LTER NIS (2005–2013)

The LTER Network Office was moved to the University of New Mexico in Albuquerque in 1996 with the promise of increasing both university space and LTER resources including those for information system efforts. The slow progress in access to data resulted in impatience for some researchers. Researchers who recognized the opportunity afforded by EML, proposed EcoTrends as a project to develop an ecological information system. The specific intention was to provide data access for data synthesis (Peters et al. 2013). Funded in 2005, EcoTrends leaders were LTER investigators who also worked for U.S. federal agencies. Fifty biome study sites distributed largely across the United States were funded to participate by NSF funding for LTER and by two U.S. Department of Agricultural agencies—the Agricultural Research Service (USDA‐ARS) and the Forest Service (USDA‐FS). Ties were maintained with NCEAS for technical support of remote data submission and ingestion into an EcoTrends data collection. The focus of EcoTrends was on producing data products for ongoing scientific analyses. LTER data managers collaborated with NCEAS and USDA partners by submitting and checking data.

The LTER Coordinating Committee approved EcoTrends as an LTER Network Information System (NIS) module. The LNO NIS‐data team was responsible for the use of the information system and of the Metacat metadata catalog in addition to the development of the project website. With LNO and NCEAS seen as a digital hub, the EcoTrends website became a familiar gateway for data submissions. LNO developers were familiar with individual data managers, the LTER IMC, and the dynamics of the LTER research community as a whole. Their attendance at LTER data manager meetings meant they were readily available and in constant contact with data managers. Together, these data groups served as a bridge between “researchers as data generators” and “researchers as data users.” An EcoTrends project coordinator proved critical to coordination and assessment of submitted data. Maintaining bilateral communications with site participants throughout the project, ensured ingestion of high‐quality data. Difficulties discovered with uploaded data were logged into a spreadsheet reporting system that was a hands‐on approach to data checking. Site‐based and cross‐site LTER scientific groups used this data to generate derived datasets that supported scientific publications. Previously such data was rarely available for discerning larger‐scale and longer‐term trends.

During this NIS phase, the community assumption that “A community data system to support scientific research is typically developed by computing professionals working for one community with funding from a single source” shifts to “A community data system to support scientific research can be led by researchers together with data workers and software engineers from one or more communities with support from one or more sources.” EcoTrends resulted in the development of an ecological information system for a number of networks of sites (Servilla et al. 2006; Peters et al. 2013). Unlike in Phase 3, where the primary leaders funded for NIS development were software engineers, the EcoTrends funded leaders were researchers who collaborated closely with software engineers and data workers. A number of new databases and communication processes were established. EcoTrends created opportunities for shared data work by researchers and data workers (Laney, Peters, and Baker 2013). EcoTrends also represented an additional workload and new partnerships at a time when LTER researchers and the NIS‐data team were considering a more comprehensive NIS architecture. NIS developers explain: “Our frustration with this [EcoTrends] labor intensive approach to providing useful ecological data gave us motivation and guidance in our development of PASTA [the Provenance Aware Synthesis Tracking Architecture]” (Servilla et al. 2016). As data managers worked on data procedures for providing quality data to EcoTrends, researchers were gaining experience with Open Data as EcoTrends made centralized data available for their immediate use.

During this period, EcoTrends datasets were being made available and NCEAS continued development of its Metacat repository. By 2009, a number of other groups were investigating and prototyping how to make data available. For instance, the Dryad effort (http://datadryad.org) was begun by library and publishing communities to provide an open data publishing platform (Vision 2010). For researchers with no repository available for their domain, it aimed to create a generalist repository that would simplify the process of linking research papers to related datasets. Dryad continues today as one of a number of generalist repository options.

### 4.5 Phase 5: An Updated LTER NIS Framework (2006–2013)

A three‐year NSF‐funded planning process by LTER to improve network‐level science began in 2004 in response to LTER reviews (Collins 2021). A model was created to address research needs that included restructuring governance and increasing cyberinfrastructure (LTER Decadal Plan 2007). Interactions with NSF failed as the agency requested a strategic plan rather than a proposed conceptual framework for social‐ecological research. An LTER Network Cyberinfrastructure Strategic Plan (2007) was not funded but was revised as an LTER NIS proposal (Servilla et al. 2016; Stafford 2021). This redesign was funded due to ARRA “stimulus funds” made available to NSF as part of government efforts to stimulate the U.S. economy. Collins (2021) noted, “Because NSF forced the LTER Network to develop a very detailed SIP [Strategic Implementation Plan], including plans for an advanced information management system to support synthesis, the LNO was poised to receive ARRA funding through NSF.” The LTER NIS ARRA proposal (2010–2014) provided a budget to support development staff and a web‐based data portal (Servilla et al. 2006, 2008).

NIS plans explained that “individual and/or collections of PASTA framework services may provide utility to sites by offering a centralized and off‐site data repository and distribution point, metadata and data congruency checks for data quality assurance and correctness, automated metadata generation and management, data access and use audits, and a source of workflow and data transformation algorithms” (Michener et al. 2011a). The architecture was designed for the upload and management of site‐generated data packages. The concept of a data package highlights the need for data submissions to include data and metadata together. Each package would be assigned a unique Digital Object Identifier (DOI). As the LTER data management community grew due to an increasing number of sites with an increasing number of data packages, the expectations and responsibilities for site data management increased as well. Bylaws were developed by the IMC as it matured as a community of practice to become a consortium with codified rules of governance (Baker, Kaplan, and Melendez‐Colom 2010). A range of viewpoints on and assumptions about data management contributed to discussions of whether minimally funded local data efforts were effective (e.g., LTER Michaels‐Powers Report 2011; Robbins 2011).

During this NIS phase, the community assumption that “A community information system can evolve into a cyberinfrastructure that provides basic access to data and metadata for a single community for discovery, access, and integration while partnering with other environmental observatory networks” shifts to an expanded and updated vision for LTER NIS where “A community information system provides advanced data capabilities that support research by establishing automated checks on data quality in addition to making data queriable, downloadable, and uniquely identified for tracking updates.” In Phase 5, the NIS approach that emerged after EcoTrends was an updated version providing advanced computing services using a service‐oriented architecture (SOA) (Servilla, O'Brien, and Costa 2013). This increased technical flexibility via improvements in the web interface, an LTER data repository, and provenance metadata for tracking data packages (Servilla and Brunt 2011; Servilla et al. 2006, 2016; Michener et al. 2011a; Waide and Kingsland 2021). The availability of ARRA funds and the readiness of the NIS‐data team explain “how” the NIS advanced during this period to become an updated, operational community data infrastructure able to support Open Science with LTER Open Data.

During this period, NSF began funding a new cyberinfrastructure program aiming to manage geoscience data. EarthCube (2011–2022, http://earthcube.org) was a 10‐year joint initiative between the NSF Directorate for Geosciences (GEO) and the Division of Advanced Cyberinfrastructure (ACI). This effort differs from the LTER and BCO‐DMO programs with their incremental growth of data infrastructure. Visioning and development for EarthCube were carried out by a number of groups of high‐performance computing specialists partnered with geoscientists each with existing data collections from within the various fields comprising the geosciences. Many individual projects were funded to provide access to the data collection. The intention was to have these collections integrated into a national technical platform when its design was complete. This program was paused in 2021 to review and reformulate thoughts about domain‐wide services that included the diverse geoscience fields (EarthCube Office and EarthCube Leadership Council 2022).

### 4.6 Phase 6: Environmental Data Initiative: A Data Management Office and Repository (2014‐Ongoing)

While the LTER NIS‐data team worked on expanding NIS into a proposed LTER cyberinfrastructure, a major change for LTER was initiated by NSF with the announcement of a re‐competition for the LTER Network Office. An open call for proposals focused on support for the LTER scientific communication and education efforts of the LTER without mention of support for data efforts. The award for this Office went to NCEAS at the University of California, Santa Barbara in 2015 for an LTER Network Communications Office, referred to as LNO today. Subsequently, an NSF call was issued for proposals to open a new Data Management Office (DMO) to support not only centralized data submissions from LTER participants but also from individuals and communities in both ecology and the environmental sciences.

A number of data managers were interested in leading an expanded DMO effort that would then be anchored at their site. Discussions within the LTER information management community and at the sites led to recognition of the need for an IMC vote to select one of these efforts to send forward. A proposal led by the University of Wisconsin‐Madison was selected by the LTER data management community and submitted. A proposal by the LNO NIS development team was also submitted to expand the LTER data repository into a multi‐community framework (sometimes referred to as updating PASTA to PASTA+) to support multiple environmental communities and individuals. At NSF's suggestion, these two efforts were merged into a single successful proposal. This grant established the two‐component Environmental Data Initiative (EDI): (1) technology services associated with the updated data repository infrastructure at the University of New Mexico at Albuquerque and (2) a Data Management Office at the University of Wisconsin at Madison, collocated with the North Temperate Lakes LTER site (Stafford 2021).

During this NIS phase, the community assumption that “A community information system can be developed to provide access to structured data and metadata for the LTER community” shifts to “An advanced community information system developed for a single community can be scaled up to include new services and a number of audiences.” After more than three decades of growth for LTER NIS and local data systems, Phase 6 began with the splitting of the LTER Network Office into two offices that reoriented LNO and established a multi‐community data effort. EDI became an independent program with funding to support ecology and environmental sciences data (Gries et al. 2021, 2023). The LTER data repository was merged into the EDI data archive with new features added. EDI today is exploring the archive of analysis‐ready data such as observations from monitoring networks. Further, EDI is a member node of the DataOne federation (Michener et al. 2011b) of data repositories. This phase makes evident how scaling up and reconfiguration of the data infrastructure was accomplished to support Open Data from ecology and the environmental sciences.

During this period, agile concepts were becoming recognized for digital infrastructure development. NSF had begun a program in the mid‐1990s for funding large‐scale science projects through Major Research Equipment and Facilities Construction (MREFC) rather than through a particular directorate, as was the case for LTER, BCO‐DMO, and EarthCube. Initially, MREFC was for major physical structures using a design approach following a “waterfall” model. This model called for design plans to be carried out over a 15‐ to 25‐year period before building of a facility would begin. The notion of “implementation” prior to “operation” was introduced in 2019 for what MREFC now was calling “large facilities” (NSF Major Facilities Guide 2019). Steinhardt (2016) observed use of spiral prototyping during work on the National Ecological Observatory Network (NEON, http://neonscience.org). NEON is a centrally administered observing network for digital data generation and distribution that was envisioned beginning in 2000, funded by MREFC in 2011 with an operational launch in 2016, and full operation in 2019 that continues today. In the international sphere, there also has been work with prototyping cycles (Chabbi and Loescher 2017; Kaltenbrunner 2017) that contributes to the loosening of digital infrastructure planning processes to allow for unforeseen pivots and actions sometimes referred to as “infrastructure growth” rather than “built infrastructure.”

## 5 Discussion

Having described the phases of incremental growth for the LTER NIS, in this section major characteristics of change are followed by overviews of the shifts in community data management assumptions and of the ongoing support for community interactions.

### 5.1 Incremental Growth Phases: Characteristics of Change and Key Findings

Establishing site‐based data management (Phase 1) and collectively envisioning network data coordination (Phase 2) are community‐defining achievements often overshadowed by advances such as the development of metadata and metadata standards (Phase 3) that support the flow of data to centralized data systems (Phase 4). As the LTER understanding of data work matured, a new data system was designed and launched from within the community (Phase 5), a system that was expanded upon subsequently in response to a call for a data management office to serve ecology and the environmental sciences (Phase 6). The complexity of growth is reflected in the number of characteristics identified below to describe each phase. These characteristics identify and name aspects that elucidate the “what” of data infrastructure, one of our research questions.

Major characteristics of the six phases in the NIS trajectory are presented in Table 3. Characteristics are grouped into three categories: scope, change element(s), and lead actor(s). There are two scope characteristics: column 2 shows the dominant arena of NIS activity and column 3 indicates the intended users of the information system. Change elements (columns 4–7) are defined earlier in the Background Section. The final three columns indicate leadership leaders in terms of responsible PIs for funded proposals (column 8), DM/Tech efforts (column 9), and major partnering contributors (column 10).

**TABLE 3:** Major characteristics of each phase identified during the growth of the LTER Network Information System.

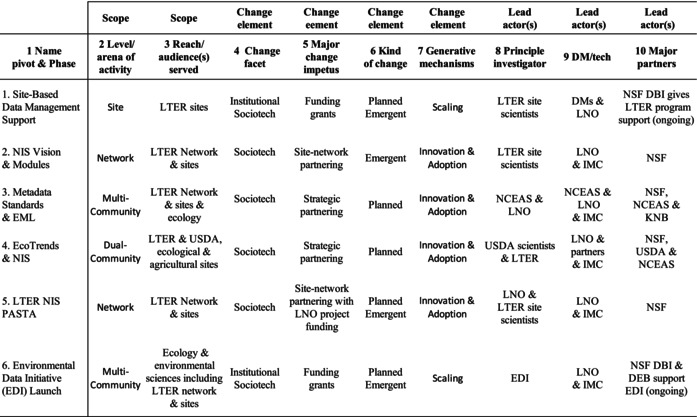

*Assessing change in each characteristic over time* reveals patterns, anomalies, and notable relations. Six key findings related to the incremental growth of data infrastructure emerge from such analysis across the phases of LTER NIS.

#### 5.1.1 Embedded Data Management

Establishing a data management role at each site was a pivotal event with long‐term ramifications. Table 3 (columns 8 and 9) shows that the data managers and the IMC play an ongoing role in NIS design in collaboration with LNO. These data‐related roles seeded the development of a workforce to support immediate site data needs and to shape longer‐term, Open Data efforts. A nuanced understanding of and respect for differences in the data and digital circumstances was stimulated by interactions with other sites. Eventually data workers developed a voice in research arenas (e.g., Michener 1986; Ingersoll, Seastedt, and Hartman 1997; Michener, Porter, and Stafford 1998; Benson et al. 2006; Michener et al. 2011a; Gries et al. 2016; Kaplan, Baker, and Karasti 2021). Embedded data managers at sites and LNOs led to researchers becoming familiar with data issues on an everyday basis. Data managers became facilitators as well as advocates for change, gaining respect as they supported research and served as both data and technology consultants at their site.

#### 5.1.2 Changing Scopes

Another key finding is that the information system scopes—both work arena level and audience shift in sync over time whether increasing or decreasing, rather than evolving in a linear fashion. The scopes of NIS during development change (columns 2 and 3), sometimes increased over the phases (Phases 1–2) while at other times they shifted to produce an interim product (Phases 3–4). The scope also contracts while regrouping in response to a community‐centric infusion of funds (Phase 5) before then expanding to serve a broader community (Phase 6).

#### 5.1.3 Awareness of Social and Technical Interdependence

A key finding for this case is that a continuing awareness of sociotechnical issues during the growth of an information system ensured community understanding and use of the information system as it changed over time. Collaborative data work calls for sensitivity when addressing changes in existing data practices and data systems. Awareness of the concept of “sociotechnical” was crucial in all six phases (column 4) ensuring high priority was given to data work and data sharing by LTER participants. We note that though EcoTrends scientists succeeded in producing a variety of published outcomes, the project's data system was not sustainable. Subsequently, in Phase 5, lead actors were from the NIS‐data team with experience with the sociotechnical aspects of LTER data practices and system design.

#### 5.1.4 Internal Partnering for Emergent Growth

Emergent growth of data management during development of a network information system was fostered by the data manager's close collaborative relationships with a combination of other data managers, researchers, and developers within the community. There are phases of emergent growth of the LTER NIS (column 6) during phases 1, 2, 5, and 6 that underscore the NIS‐data team work. In these phases, the leaders were situated within the LTER itself rather than external to the community (column 9). Internal relations within the community take a variety of forms such as working groups, workshops, and a small group of data managers working with NIS developers on a targeted topic, referred to as “tiger teams.”

#### 5.1.5 External Partnering for Planned Growth

Another key finding is that intermittent work with external partners (column 10) was critical to the growth of the community and the community's information system. Partners contributed to developing approaches, procedures, and services for work with data and technology. Their work is detailed in Section 4 by phase. In addition, the NSF funding agency played a significant role as an institutional partner in communicating with LTER participants and guiding research arrangements.

#### 5.1.6 Scaling Prompts From External Sources

Major increases in audience for data management and computing efforts were spurred by external interventions in the form of institutional mandates associated with funding. Working with community members, NSF with great vision and remarkable insight established LTER. Dramatic change in research arrangements was introduced in Phase 1 via a mandate for each site to designate a data management role tied to their long‐term funding. The role was made explicit in NSF proposals, yet loosely defined, leaving the flexibility for it to evolve at the project level. Another example of a scaling prompt was NSF's call for proposals in Phase 6 to create a national data initiative that served LTER and multiple other communities. NSF funding supported expansion of the LTER NIS from a single‐community information system to its current state with EDI. Scaling prompts are reflected in the audience served (column 3), the institutional change (column 4), and the impetus enabling change (column 5). Program and project funding represents a significant generative mechanism for scaling data management and data system activities (column 7).

*These six key findings from assessing change during the NIS incremental growth over time* appear to sidestep some of the “path dependence” or “lock‐in” associated with traditional major digital endeavors with more hierarchical structures (Edwards et al. 2007; Hirsch, Ribes, and Inman 2022). For example, in Phase 2 sites were successful in making local data accessible and prototyping network modules within the community. In contrast, during Phase 3 sites shifted focus to work with external technology‐oriented partners on an ecological metadata standard. Yet, lock‐in did not occur when these partners presented metadata generation tools. The tools were explored but typically not adopted at LTER sites since they did not align with the diverse local data workflows.

### 5.2 Changing Assumptions Relating to Data Management

Changes in long‐held perceptions or operating assumptions about data management in the LTER community over the time period of NIS development (see Section 4) are summarized in Table 4. They show the assumptions becoming more complex over time as data management itself becomes more complex.

**TABLE 4:** Transformation in LTER assumptions about digital data management by phase.

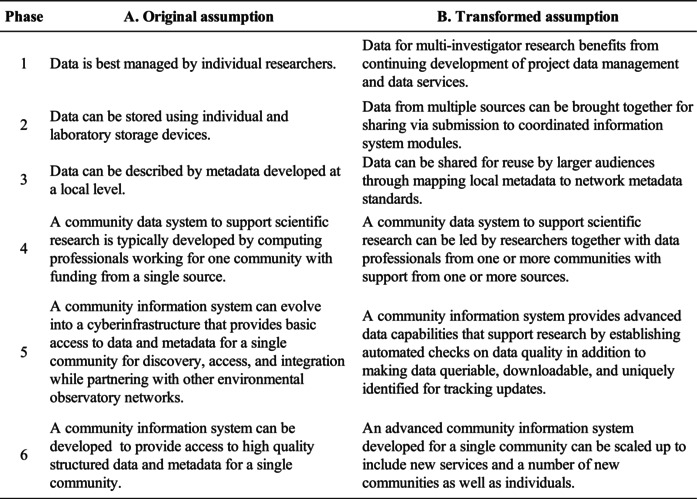

Research has shown that unnoticed community and cultural assumptions create barriers to change (e.g., Gitelman 2013; Edwards et al. 2011). A literature survey of large‐scale architectures examined the need for more widespread understanding of changing data practices across communities (Uludag et al. 2018). This includes providing time for the development of what is described as agileness of “culture and mindset.”

Stepwise change in community understanding is evident in the LTER case. The community's uptake and conceptual shifts over the extended period suggest that the LTER NIS incremental growth provided the time required for wide‐spread discussion and understanding of data practices across the network that contributed to changing assumptions about data management. There is ongoing socialization and a form of response that has been called “assumptions wrangling” (Cutcher‐Gershenfeld 2018). The many year time period of each phase provided ample opportunities for community members to witness and experience digital prototypes and products as outcomes or indicators of success.

### 5.3 Supporting LTER Community Interactions

The LTER program communication structure is key to making use of the time provided by the evolution of NIS. This structure (Figure 1) is identified as a contextual condition of the LTER Network (see Section 2.5.2). Its support for continuing interactions is crucial to collaborative processes involving LTER data‐oriented, technology‐oriented, research‐oriented, and policy‐oriented participants within and across work arenas. Collaboration is integral to the interdisciplinarity of ecology and the value of long‐term ecological projects in terms of their potential to help participants learn to collaborate has been recognized (e.g., Mauz et al. 2012). In support of the LTER community's collaborative activities, each component in the communication structure actively processes and shares information while making decisions in both planned and improvisational ways. The non‐hierarchical nature of Figure 1 suggests that actions are negotiated, considered not just from one perspective but reconsidered and informed by many participants across the components. Bringing these components together distributes power and ties together a diversity of perspectives that stimulates discussion and prompts reflection. This visual shows the extent of potential interactions and agency as well as the messiness inherent to data and infrastructure work in the LTER. Such extensive interactions are time‐consuming and require patience, yet they can have positive impacts on research coordination together with management of knowledge, data, and the program. These are features identified as contributing to development and sustainability of large‐scale enterprises (Uludag et al. 2018). The structure creates many potential points of learning, adaptation, and trust‐building (Gunderson and Holling 2002). Such an environment requires continuing attention to balancing the predictable and unpredictable. Co‐learning, or learning as a group, is fostered by such an environment. Creativity, together with learning, is fostered by such an environment. Engeström (2001) reminds us that creativity does not only happen inside people's heads.

The LTER approach exhibits many “bottom‐up” insights into how technology can support effective and large‐scale digital efforts. This case with its multi‐level communications and work arenas, however, may be recognized more fully as encompassing multiple approaches to growth given its development in phases with differing characteristics and many data work arenas (Table 3). Figure 1 suggests that change in the LTER NIS occurs (a) bottom‐up with a local narrative that is maintained and promoted informally at sites, (b) middle‐out via lateral or partnering to address change with a network narrative that promotes updates and revisions, and (c) top‐down with a meta narrative that may be community promoted or formally mandated. LTER NIS Phases 1 and 6 in particular reflect top‐down interventions that do not disturb scientific research plans. These top‐down interventions do, however, seed what occurs with NIS prototyping (Phase 2), with NIS redesign (Phase 5), and with NIS evolution into a multi‐community system (Phase 6).

The Phase 6 pivot that resulted in reorganization of LNO effectively separated direct oversight of the LTER NIS growth from the governance of LNO and LTER researchers (Figure 1). This represents a major shift from its earlier launch and growth environment for data management, which was nested within the LTER research community. The independent EDI organizational structure is now more similar to other large‐scale digital infrastructures, many of which are led by computing, technology, and/or software engineers unlike the early NIS efforts with a number of participants having a long‐term view of data practices. There are issues to consider with these new arrangements: (a) the separation of the network information system from direct governance by the community generating the data; (b) the potential distancing of local data managers as contributors to the development of network data infrastructure which in turn impacts local data and design capacities; and (c) the impact on identity and leadership for LTER IMC from which emerged a number of EDI leaders. Continuing study of EDI as a national data management center and as an environmental sciences platform, including its data archive, would be informative. Will the LTER community assumptions continue to be transformed? What pivots and opportunities will influence the EDI trajectory as it becomes one of a growing number of national data offices?

## 6 Conclusion

The LTER case illustrates a research‐driven ecology program that fostered the development of site‐based data management and local data systems prior to designing, launching, sustaining, and scaling up a centralized network information system. This case provides an approach to Open Data that has proven effective in its ability to implement data management in addition to supporting the evolution of data infrastructure. The three research questions posed earlier are discussed below with the LTER case in mind and are followed by final remarks.

### 6.1 The How, What and When of Data Infrastructure

#### 6.1.1 How Does the Growth of Digital Data Infrastructure Occur Over Time?

The Incremental Growth Model operationalizes the notion of “infrastructuring,” creating an analytical tool offering specificity useful for analysis. Considering the LTER NIS as a multi‐phase trajectory foregrounds the concept of infrastructure growth as an ongoing process from an initial vision through to continuing use. Drawing on Science and Technology Studies and Infrastructure Studies concepts of “continuing design” and “infrastructure growth,” this long‐term ethnographic story of a community information system could be framed as a biography of data infrastructure (Pollock and Williams 2010). Instead, the Incremental Growth Model incorporates some empirical contributions from Organizational Change Research, including the concepts of contextual conditions, pivotal events, and major phase characteristics. By merging the concepts from these fields, the model integrates the variety of often taken‐for‐granted activities into a story that highlights emergent opportunities and adaptive responses to change in the trajectory of an information system.

Given the incremental growth of LTER NIS, time was available for the interactions that resulted in the building of personal relationships and trust. The pace of NIS development allowed time for understandings to grow and diffuse across the community, thereby shifting assumptions about digital data management. The start and end of each phase served as points of community discussion where experiential learning and shared decision‐making occurred. Recognition of individual phases and their outcomes stimulated periodic reflections on successes among participants while reaffirming data‐related work as a long‐term mission that required “staying the course” with nudges for those within the community who were resisting or lagging in their data practices. Stepwise development also enabled data workers to grow into key actors in the digital environment. Indeed, a number of site‐based data managers and LNO NIS developers who participated in earlier phases of NIS growth were active in the scaling up to EDI.

#### 6.1.2 What Characteristics and Data Workers Are Associated With the Evolution of an Information System?

Major characteristics summarized in Table 3 and discussed in Sections 4 and 5 describe the data work associated with each phase of the LTER NIS trajectory. Insights into infrastructure‐making will increase as major characteristics and categories of infrastructure in a variety of contexts are identified in long‐term studies. The many processes used and paths occurring in data infrastructure growth are leading to collections of empirical studies (Harvey, Jensen, and Morita 2017) and to calls for more comparative analysis (Henfridsson and Bygstad 2013; Miller 2018). In seeking explanations that take into account both the dynamics of digital environments and the various circumstances shaping their evolution, Henfridsson and Bygstad (2013) conclude “more knowledge about what drives digital infrastructures would be highly valuable for managers and IT professionals confronted by the complexity of managing them.”

Many roles are emerging to carry out data work today. A notable aspect of the LTER NIS evolution is the development of a data workforce within the community. Data management is identified as a contextual condition of the LTER network (see Section 2.5.2). Embedded data management is described in the first phase of the LTER NIS trajectory and highlighted as one of six key findings in analysis of the NIS evolution (see Section 5.1.1). LTER data workers keep data management and local research needs in sync, thereby minimizing disruptions to research often caused by new technologies and data requirements. Although the phases in this case highlight a variety of actors, the role of data managers for LTER remains key as they serve as liaisons, designers, implementers, and sustainers of data‐related work (Millerand and Baker 2010, 2020).

#### 6.1.3 When Does the Growth of Digital Data Infrastructure Begin?

The LTER information system trajectory is incremental and cumulative, making it difficult to pinpoint a single time at which infrastructure begins. Each pivot signals the beginning of change and demarks a new phase. Each phase sets the stage for subsequent phases. In the 1980s (Phase 1) when the internet was not widely available, the NSF funding agency mandate establishing data management operationalized a role that facilitated change in community data practices and in data system developments. When funding became available in 2008 Phase 5 for an LTER NIS‐data team to revise the NIS Framework, the LTER community had matured to recognize the LTER NIS as sustainable infrastructure providing access to LTER data. And Phase 5 was key to scaling up in Phase 6 when the support for the LTER NIS shifted from direct reporting within the LTER to become EDI interacting with LTER as an independently funded data entity.

### 6.2 Final Thoughts

The LTER case illustrates a way of working with data and data systems on a time scale that accommodates collaborative activities and the incremental growth of data infrastructure. The evolution of data‐related arrangements was facilitated by the continuity afforded to the LTER program by long‐term support from NSF. Researchers traditionally trained and experienced in using their own data were exposed, over their years with LTER, to the work associated with data sharing that led to Open Data. Study of the six phases of the LTER NIS trajectory has revealed the many ways participants contribute to the growth of data infrastructures. The Incremental Growth Model of data infrastructure captures the NIS trajectory by focusing on three distinct features: (1) contextual conditions, (2) sequential phases initiated by pivotal events, and (3) major characteristics for each phase.

Open Science depends upon Open Data, that is, high‐quality data widely available for reuse. The LTER case provides specifics regarding data arrangements and struggles associated with data infrastructure growth. This longitudinal study brings together insights into the incremental growth of a network information system together with the cultural growth of a research community steadfast in addressing changes in both ecological systems and digital systems. The LTER NIS trajectory culminated in a Data Management Office and a digital hub with a data repository. Open Data requires data repositories available to ingest, store, and package data for access, discovery, and reuse yet critical, long‐term studies of how to develop data infrastructure are rare.

The LTER case is an example of successful data infrastructure growth. As experience with digital systems grows alongside understandings of how “long‐term matters in collaborative development” (Karasti, Baker, and Millerand 2010), we can reflect on the four decades of continuing design leading to the opening of the EDI national data management office and data repository for the environmental sciences. Early programs did not consider sociotechnical development nor did they prioritize local data managers. The scaling up of LTER NIS to EDI was configured to ingest data not only from gateway systems but also from individual investigators. Further, unlike early design approaches, the LTER NIS illustrates continuing design as it contributes to shifts in community assumptions, draws on effective communication structures, and facilitates growth of a data workforce aware of sociotechnical complexity. EDI will continue to address sustainability challenges and new challenges that may benefit from further collaboration with social scientists, historians, and organizational change researchers as LTER enters its fifth decade.

In contrast to the technological focus of many studies, this LTER study highlights the importance of taking a close look at the variety of characteristics that change and define the phases of a data infrastructure trajectory. The NIS growth can be viewed as fragmented and inefficient especially by those whose experience in practice and/or in theory is derived from cases following largely linear, technical plans rather than from cases of complex data ecosystems with interdependent social, technical and institutional facets at multiple levels. We argue that the LTER data work fits within a broader definition of success than is typical for data infrastructure, one that strengthens a community's data literacy, data management, and its local data workforce by continuously attending to communication, collective experience, and change. Supported by development of local data capacities, an ecological community's operational network data infrastructure was transformed into a national data management office and repository for the ecological and environmental sciences. The LTER case of data infrastructure can be described as an organizational achievement attained through long‐term incremental growth.

## Author Contributions

**Karen S. Baker:** conceptualization (lead), formal analysis (lead), investigation (lead), methodology (lead), visualization (lead), writing – original draft (lead), writing – review and editing (equal). **Florence Millerand:** conceptualization (supporting), methodology (supporting), visualization (supporting), writing – review and editing (supporting).

## Conflicts of Interest

The authors declare no conflicts of interest.

## Data Availability

The authors have nothing to report.
